# Restless legs syndrome induced by fexofenadine/pseudoephedrine

**DOI:** 10.1002/jgf2.338

**Published:** 2020-06-15

**Authors:** Hiroaki Nishioka, Yohei Kanzawa

**Affiliations:** ^1^ Department of General Internal Medicine Kobe City Medical Center General Hospital Kobe Japan; ^2^ Department of General Internal Medicine Akashi Medical Center Akashi Japan

**Keywords:** antihistamine, fexofenadine, pseudoephedrine, restless legs syndrome, sleep disorder

## Abstract

Antihistamines are known risk factors for restless legs syndrome (RLS). However, reports on RLS associated with fexofenadine or its combinations are rare. Here, we report a 30‐year‐old woman with RLS that was induced by fexofenadine/pseudoephedrine. She had been taking fexofenadine/pseudoephedrine for three months and felt a strong urge to move her legs at night, which was relieved by movement. Her condition improved by taking pramipexole, which she discontinued subsequently because of dizziness. One month later, she quitted taking fexofenadine/pseudoephedrine, after which her symptoms disappeared a week later. This case study demonstrates that RLS can be induced by fexofenadine/pseudoephedrine and we should always consider the possibility of drug‐induced RLS.

## 1 INTRODUCTION

Restless legs syndrome (RLS) is a sensorimotor disorder characterized by an unpleasant or uncomfortable urge to move the legs. It occurs during periods of rest or inactivity, particularly at night, and is transiently relieved by moving the legs. The pathophysiology of RLS remains poorly understood; however, several medications are known to exacerbate existing RLS or possibly precipitate RLS. These include antihistamines^1^, ^2^, ^3^, dopamine antagonists^4^, antidepressants^5^, and lithium^6^. Fexofenadine is an antihistamine and is commonly used for treating allergy symptoms throughout the world. However, reports on RLS associated with fexofenadine or its combinations are rare. We herein report a case of RLS that was induced by fexofenadine/pseudoephedrine.

## 2 CASE REPORT

A 30‐year‐old woman presented to our hospital with a one‐month history of discomfort in her legs and a strong urge to move them at night, which led to a sleep disorder. Her symptoms were transiently relieved by moving her legs. Her medical history included asthma and allergic rhinitis. She had been taking fexofenadine/pseudoephedrine (60 mg/120 mg) twice a day for seasonal allergic rhinitis, which was prescribed by another clinic for three months. She did not take any other drug, supplement, or alcohol. She smoked five cigarettes and drank five cups of coffee per day. She was not pregnant. These medical histories were obtained by face‐to‐face interview by a physician.

On physical examination, the patient's blood pressure was 102/72 mm Hg; heart rate, 61 beats/minute; respiratory rate, 12 breaths/minute; and body temperature, 36.5°C. Examination of her respiratory, cardiovascular, and gastrointestinal systems did not reveal any remarkable findings. Leg edema and rash were not observed.

No abnormal neurological findings of the legs were recognized, including Achilles tendon reflex. Laboratory examination revealed a white blood cell count of 4500 cells/μL with 55% neutrophils, a hemoglobin level of 12.7 g/dL with mean corpuscular volume of 95, a platelet count of 13.9 × 10^4^/μL, blood urea nitrogen of 12.9 mg/dL, creatinine of 0.64 mg/dL, a C‐reactive protein level of 0.01 mg/dL, and a ferritin level of 46 ng/mL.

We suspected her of RLS and initiated prescription oral pramipexole at 0.125 mg/day. Her RLS symptoms diminished; however, she could only take it for 3 days because of the development of severe dizziness. Her RLS symptoms continued. One month later, her doctor at the other clinic terminated her prescription of fexofenadine/pseudoephedrine as her seasonal allergic rhinitis had improved. After which her RLS symptoms reduced, and one week later, the symptoms completely disappeared. Since then, she has not taken fexofenadine/pseudoephedrine and her RLS symptoms have not recurred for some years. We think that she underwent RLS induced by fexofenadine/pseudoephedrine.

## 3 DISCUSSION

The clinical course of this patient shows that RLS can be induced by fexofenadine/pseudoephedrine. The pathophysiology of RLS remains unclear; however, both central and peripheral nervous system abnormalities are considered associated with RLS development. The central nervous system alterations in patients with RLS include central iron stores, dopamine function, thalamic function, the opioid system, and the glutamatergic system. Histamine is one of the neurotransmitters in the brain and may act as both a neuromodulator and a classical transmitter. First‐generation antihistamines, particularly those that are centrally acting (sedating), have sometimes reported to be associated with RLS development.^1^, ^2^, ^3^ However, reports on RLS with second‐generation antihistamines are rare. One of the differences between first‐ and second‐generation antihistamines is the value of H1 receptor occupancy in the brain.^7^ The high value of first‐generation antihistamines is considered associated with RLS development. Fexofenadine is a commonly used second‐generation antihistamine and is classified as a nonsedating antihistamine based on the low value of H1 receptor occupancy in the brain.^7^ Reports on RLS associated with fexofenadine have not been described. However, our patient clearly showed that RLS was induced by fexofenadine/pseudoephedrine. In the case of taking fexofenadine/pseudoephedrine, the blood concentration of fexofenadine has been reported to be approximately one and a half times as much as taking the same dose of fexofenadine alone.^8^ In this case, this increased concentration might have affected RLS development.

The peripheral and autonomic nervous system has also been implicated in the pathogenesis of RLS.^9^ Abnormalities of microvascular function in the legs have also been demonstrated in patients with RLS, including altered leg intramuscular blood flow, peripheral hypoxia, and altered endothelial function.^10^ Pseudoephedrine is a sympathomimetic amine and acts on adrenergic receptor systems, which might affect peripheral nervous system or microvascular function in the legs. Restless legs syndrome cases associated with pseudoephedrine have not been reported; however, its combination with fexofenadine might have affected RLS development in our case.

In conclusion, RLS can be induced by fexofenadine/pseudoephedrine. Restless legs syndrome is a clinical diagnosis mainly made by taking the history and a physical examination of the patient. This case study shows that we should always consider the possibility of drug‐induced RLS.

## CONFLICT OF INTEREST

The authors have stated explicitly that there are no conflicts of interest in connection with this article.

## INFORMED CONSENT

We obtained informed consent from the patient for publication of this case report.
